# Supplementary material for: Indicators of retention in remote digital health studies: a cross-study evaluation of 100,000 participants
Source: NPJ Digit Med. 2020 Feb 17;3:21. doi: 10.1038/s41746-020-0224-8 (PMC7026051; doi:10.1038/s41746-020-0224-8)

Supplementary Table 1: Comparison of median survival time (with 95% confidence intervals) across studies using two analytical approaches with and without censoring.s

		No censoring		With right censoring	
**Strata**	**N**	**median**	**95% CI**	**median**	**95% CI**
SleepHealth	12850	2	2-2	2	2-2
Brighten	875	26	17-33	26	18-33
Asthma	4666	12	11-13	12	11-13
ElevateMS	605	7	5-10	7	5-10
mPower	6908	4	4-5	4	4-4
Phendo	7802	4	3-4	3	3-4
MyHeartCounts	18671	9	9-9	8	8-9
Start	42237	2	2-2	2	2-3

Supplementary Table 2: Comparison of median survival time (with 95% confidence intervals) across gender(Female vs Male) stratified by studies using two analytical approaches with and without censoring.

		No censoring		With right censoring	
**Strata**	**N**	**median**	**95% CI**	**median**	**95% CI**
gender=Female|SleepHealth	3659	2	2-2	2	2-2
gender=Female|Brighten	681	25	16-33	26	16-34
gender=Female|Asthma	993	9	7-12	9	7-11
gender=Female|ElevateMS	244	35	26-49	39	26-53
gender=Female|mPower	1998	4	4-5	4	3-5
gender=Female|Phendo	7532	4	3-4	3	3-4
gender=Female|MyHeartCounts	1320	12	11-13	12	11-13
gender=Female|Start	32067	2	2-2	2	2-3
gender=Male|SleepHealth	8898	2	2-2	2	1-2
gender=Male|Brighten	194	30	12-44	30	12-44
gender=Male|Asthma	1516	7	5-9	7	6-9
gender=Male|ElevateMS	85	26	16-57	45	26-75
gender=Male|mPower	4886	4	4-5	4	4-4
gender=Male|MyHeartCounts	5641	14	13-14	14	13-15
gender=Male|Start	10170	2	2-2	3	2-3

Supplementary Table 3: Comparison of median survival time (with 95% confidence intervals) across age groups stratified by studies using two analytical approaches with and without censoring.

		No censoring		With right censoring	
Strata	N	median	95% CI	median	95% CI
age_group=18-29|SleepHealth	4063	2	2-2	1	1-1
age_group=18-29|Brighten	438	18.5	13-31	19.5	13-31
age_group=18-29|Asthma	1088	4	3-4	3	2-4
age_group=18-29|ElevateMS	62	1	1-3	1	1-2
age_group=18-29|mPower	2139	2	1-2	1	1-1
age_group=18-29|Phendo	4145	3	3-4	3	3-4
age_group=18-29|MyHeartCounts	390	2	2-4	2	1-3
age_group=18-29|Start	23537	2	2-2	2	2-2
age_group=30-39|SleepHealth	3558	2	2-2	2	1-2
age_group=30-39|Brighten	220	35	20-49	35	20-49
age_group=30-39|Asthma	699	8	6-10	8	6-11
age_group=30-39|ElevateMS	151	6	2-12	5	2-12
age_group=30-39|mPower	1251	3	2-3	2	2-3
age_group=30-39|Phendo	2701	4	3-5	4	3-4
age_group=30-39|MyHeartCounts	505	3	2-4	3	2-6
age_group=30-39|Start	10200	2	2-3	3	3-4
age_group=40-49|SleepHealth	2574	2	2-3	2	2-2
age_group=40-49|Brighten	129	39	12-65	39	12-68
age_group=40-49|Asthma	362	12	9-19	13	9-21
age_group=40-49|ElevateMS	162	8	5-13	7.5	5-13
age_group=40-49|mPower	898	5	4-6	5	4-6
age_group=40-49|Phendo	616	4	3-6	4	3-5
age_group=40-49|MyHeartCounts	253	8	5-10	7	4-10
age_group=40-49|Start	5236	2	2-2	3	2-3
age_group=50-59|SleepHealth	1363	3	3-4	3	2-4
age_group=50-59|Brighten	61	42	14-83	42	14-83
age_group=50-59|Asthma	228	32.5	19-53	36	20-56
age_group=50-59|ElevateMS	126	12	7-24	11	7-24
age_group=50-59|mPower	927	9	7-10	10	8-12
age_group=50-59|Phendo	19	1	1-30	1	1-73
age_group=50-59|MyHeartCounts	188	6	4-10	6	4-10
age_group=50-59|Start	2204	2	1-2	3	2-4
age_group=60+|SleepHealth	833	4	3-6	4	3-6
age_group=60+|Brighten	27	1	1-25	1	1-25
age_group=60+|Asthma	135	57	40-70	57	39-70
age_group=60+|ElevateMS	68	18	8-53	20	8-75
age_group=60+|mPower	1588	15	13-17	15	13-19
age_group=60+|Phendo	3	1	1-1	1	1-1
age_group=60+|MyHeartCounts	216	9.5	7-17	11	7-26
age_group=60+|Start	1046	1	1-1	2	1-2

Supplementary Table 4: Comparison of median survival time (with 95% confidence intervals) across disease of interest to the study(True/False) stratified by studies using two analytical approaches with and without censoring.

		No Censoring		With right censoring	
**Strata**	**N**	**median**	**95% CI**	**median**	**95% CI**
caseStatus=FALSE|SleepHealth	12269	2	2-2	2	1-2
caseStatus=FALSE|Asthma	86	12	6-20	12.5	5-20
caseStatus=FALSE|ElevateMS	132	1	1-2	1	1-1
caseStatus=FALSE|mPower	4049	3	3-3	3	2-3
caseStatus=FALSE|MyHeartCounts	15666	10	9-10	9	9-9
caseStatus=TRUE|SleepHealth	500	16.5	14-20	16.5	14-20
caseStatus=TRUE|Asthma	4249	14	13-15	14	13-15
caseStatus=TRUE|ElevateMS	473	12	8-14	11	7-15
caseStatus=TRUE|mPower	1896	15	13-17	15.5	14-19
caseStatus=TRUE|MyHeartCounts	1009	10	9-11	10	9-11

Supplementary Table 5: Comparison of median survival time (with 95% confidence intervals) across participants that were clinically referred(True/False) stratified by studies using two analytical approaches with and without censoring. *After right censoring, the retention in the clinically-referred cohort in the mPower study did not drop below 50%. Therefore the lowest retention time(76 days) for 52.9% retention is shown.

		No censoring		With right censoring	
**Strata**	**N**	**median**	**95% CI**	**median**	**95% CI**
clinicalReferral=FALSE|ElevateMS	469	4	2-6	4	2-6
clinicalReferral=FALSE|mPower	6836	4	4-4	4	4-4
clinicalReferral=TRUE|ElevateMS	136	25.5	17-55	26	17-56
clinicalReferral=TRUE|mPower	72	58.5	51-83	> 76*	55-NA

Supplementary Table 6: Change in median survival time (with 95% CI) based on the minimum number of days(N = 1-32) a subset of participants continued to use the study apps

Study	minimum duration	median	95% CI
SleepHealth	1	2	2-2
Brighten	1	26	17-33
Asthma	1	12	11-13
ElevateMS	1	7	5-10
mPower	1	4	4-5
Phendo	1	4	3-4
MyHeartCounts	1	9	9-9
Start	1	2	2-2
SleepHealth	2	6	6-7
Brighten	2	71	63-81
Asthma	2	23	21-24
ElevateMS	2	26	21-35
mPower	2	13	13-14
Phendo	2	16	15-17
MyHeartCounts	2	12	12-13
Start	2	16	16-17
SleepHealth	4	13	12-13
Brighten	4	75	66-83
Asthma	4	27	25-29
ElevateMS	4	35	26-46
mPower	4	20	19-21
Phendo	4	25	24-27
MyHeartCounts	4	14	14-14
Start	4	21	20-22
SleepHealth	8	21	20-22
Brighten	8	81	72-83
Asthma	8	33	31-35
ElevateMS	8	48	37-57
mPower	8	28	26-29
Phendo	8	36	34-38
MyHeartCounts	8	17	16-17
Start	8	29	28-29
SleepHealth	16	36	34-38
Brighten	16	83	82-83
Asthma	16	44	42-46
ElevateMS	16	69	57-75
mPower	16	42	41-44

Phendo	16	49	47-51
MyHeartCounts	16	35	34-36
Start	16	35	35-36
SleepHealth	32	60	57-62
Brighten	32	84	83-NA
Asthma	32	64	61-66
ElevateMS	32	77	75-81
mPower	32	58	56-60
Phendo	32	65	63-67
MyHeartCounts	32	60	59-62
Start	32	52	51-53

Supplementary Table 7: Proportion of missingness in the selected demographics across all the eight studies

**Demographics Variable** %(N)	**Asthma**	**Brighten**	**ElevateMS**	**mPower**	**MyHeartCounts**	**Phendo**	**SleepHealth**	**Start**
Age	41.24 (1763)	0.8 (7)	5.95 (36)	1.53 (106)	91.47 (16669)	0.64 (48)	2.96 (378)	0.03 (14)
Gender	41.31 (1766)	0.8 (7)	45.62 (276)	0	83.1 (15145)	0	1.66 (212)	0
Race	23.42 (1001)	0.8 (7)	44.79 (271)	0.46 (32)	74.25 (13532)	0.03 (2)	58.41 (7459)	NA
State	62.83 (2686)	7.19 (63)	45.45 (275)	7.09 (490)	41.55 (7572)	53.32 (4016)	100 (12770)	44.84 (19148)

Supplementary Table 8: Summary of participants' daily study app usage behavior and demographics across five distinct engagement clusters

Cluster Characteristics	dedicated	high	moderate	sporadic	abandoners
	**C1**	**C2**	**C3**	**C4**	**C5***
**N**	3,369	4,847	14,339	16,607	55,452
**Proportion of Participants(%)** (median ± IQR)	3.5 ± 2.5	6 ± 3.1	12.5 ± 2.9	21 ± 9.6	54.6 ± 17.3
**Duration in study app** (median ± IQR)	81 ± 22	53 ± 37	18 ± 21	19 ± 25	1 ± 1
**Regularity of app usage in the first 84 days(%)** (median ± IQR)	96.4 ± 26.2	63.1 ± 44.1	21.4 ± 25	22.6 ± 29.8	1.2 ± 1.2
**Days Active in study app** (median ± IQR)	39 ± 39	24 ± 13	9 ± 5	4 ± 3	1 ± 1
**Days between activity** (median ± IQR)	2 ± 1	2 ± 1	2 ± 1	5 ± 7	NA
**Age group - (median ± IQR)**					
18-29	20.5 ± 29.2	28.3 ± 31.7	33.7 ± 35.7	33 ± 26	45.1 ± 18.6
30-39	21.5 ± 12.4	26.5 ± 7.3	27.8 ± 4.6	28 ± 8.1	27.8 ± 7.7
40-49	15.7 ± 5.4	16 ± 9.6	14.2 ± 10.8	15.5 ± 4.3	13.6 ± 3.3
50-59	12.4 ± 12.5	12.2 ± 9.1	10 ± 8.8	11.2 ± 8.5	8 ± 4.9
60+	17.2 ± 29.3	15.1 ± 11.7	11.7 ± 11.3	6.3 ± 9.2	5.1 ± 6.6
**Race/Ethnicity (median ± IQR)**					
Non-Hispanic White	82.8 ± 4.8	78.4 ± 9.9	77.6 ± 9.3	71.6 ± 9.3	73.6 ± 8.5
Hispanic/Latinos	6.5 ± 3.6	7.5 ± 5.4	10.3 ± 6.6	8.8 ± 5.4	9.3 ± 8.1
African-American/Black	5.3 ± 3.5	3.3 ± 1.6	3.2 ± 2.8	5.3 ± 3.2	4.4 ± 3.5
Asian	3.7 ± 2.8	4.8 ± 2.1	5.3 ± 2.6	8 ± 3.6	6 ± 2.5
AIAN	0.7 ± 0.5	0.6 ± 0.4	0.3 ± 0.2	0.8 ± 0.1	0.4 ± 0.3
Hawaiian or other Pacific Islander	0.6 ± 0.3	0.5 ± 0.6	0.3 ± 0.7	0.6 ± 0.6	0.5 ± 0.4
Other	3.5 ± 1.8	3.8 ± 2.7	5 ± 3.1	4.5 ± 2.3	6 ± 2.6

Supplementary Figure 1: Sensitivity analysis comparing the participant retention across **a)** age groups including participants for whom age was not available and **b)** gender including participants with missing gender. For all survival curves, the shaded region shows the 95% confidence limits based on the survival model fit.s

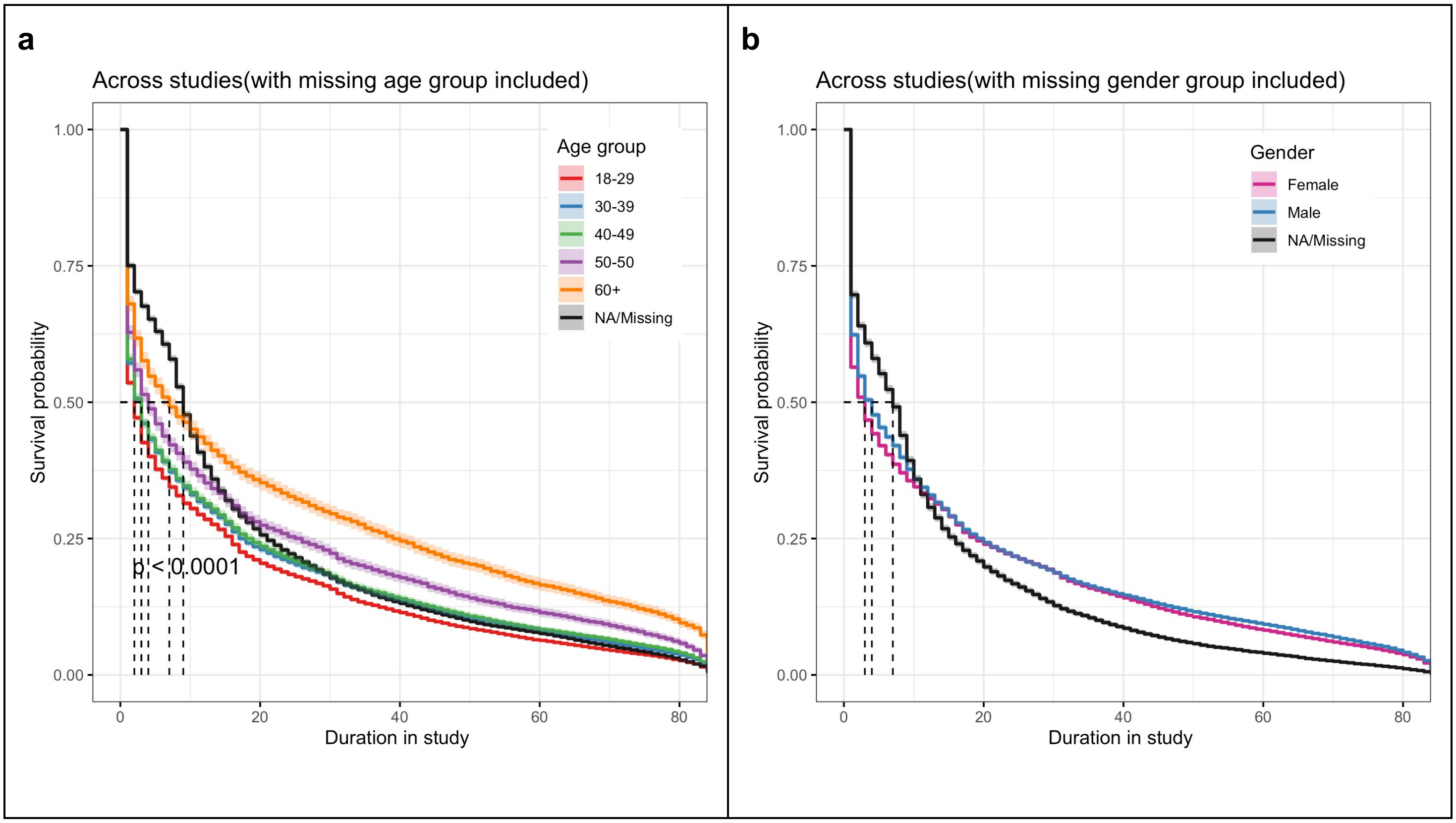

Supplementary Figure 2: Comparison of within-cluster variations across studies using different cluster sizes (N=1-10) for K-means clustering. Seven out of eight studies showed the within-cluster variation to be minimum by partitioning the daily app usage into four clusters.

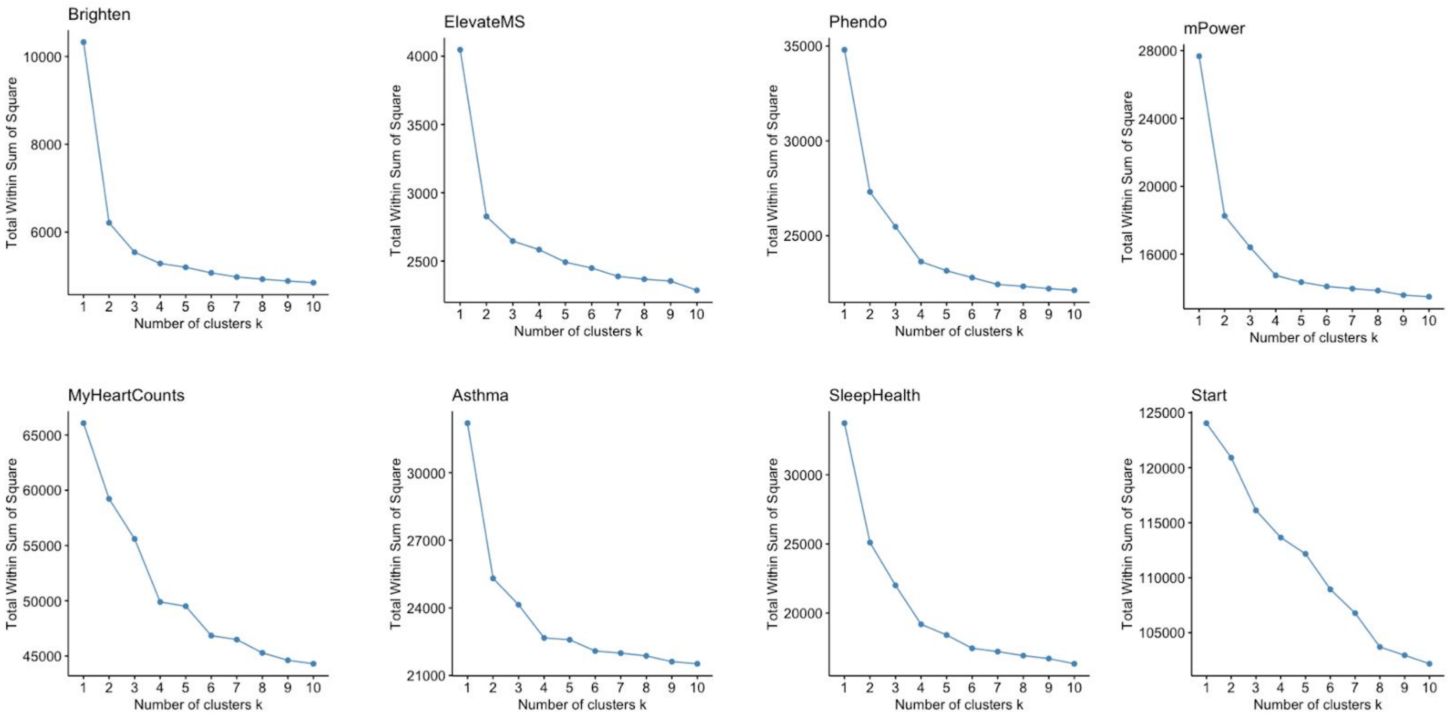

Supplement: Supplementary file 1 — Supplementary Material [file 41746_2020_224_MOESM1_ESM.pdf]
